# Risk factors and coronary artery outcomes of coronary artery aneurysms differing in size and emergence time in children with Kawasaki disease

**DOI:** 10.3389/fcvm.2022.969495

**Published:** 2022-09-09

**Authors:** Jie Liu, Qiaoyu Yue, Suyuan Qin, Danyan Su, Bingbing Ye, Yusheng Pang

**Affiliations:** Department of Pediatrics, First Affiliated Hospital, Guangxi Medical University, Nanning, China

**Keywords:** Kawasaki disease, coronary artery aneurysms, intravenous immunoglobulin, risk factor, regression

## Abstract

Coronary artery aneurysm (CAA) is a serious cardiac complication arising from Kawasaki disease (KD) and is becoming the leading cause of acquired heart disease in children. The aim of this study was to determine the potential risk factors associated with coronary artery aneurysms (CAAs), which differ in size and emergence time, and track its regression within 3 years of onset. The laboratory data, clinical features, and coronary artery outcomes of patients, who were diagnosed with KD and received treatment from January 2003 to January 2019 were retrospectively analyzed. A total of 484 pediatric patients with KD were examined during the study period. Among them, 130 (26.9%) presented with CAA, including mid- to large-sized CAA in 38 patients (7.9%) and *de novo* CAA after intravenous immunoglobulin (IVIG) treatment in 22 patients (4.5%). Albumin-to-globin (A/G) ratio was significantly negatively associated with the absolute internal diameter of coronary artery at 1 month of onset and may be used as a predictor of mid- to large-sized CAA development in patients with KD. The area under the receiver operating characteristic curve was 0.637 (95% confidence interval: 0.551–0.724), and a cutoff of 1.32 yielded a sensitivity and specificity of 79 and 49%, respectively, for predicting mid- to large-sized CAA development. *De novo* CAA after IVIG may lead to an increased risk of developing progressive CAA [13 (59.1%) of 22 vs. 31 (28.7%) of 108; *P* = 0.006] and had significantly greater changes in both the magnitude of CAA dimension variation and maximum z-score of the coronary arteries at 2 and 4 weeks and then 3 months after onset (*P* < 0.001). Kaplan–Meier survival analysis revealed that the estimated median time of aneurysm persistence was significantly higher in the progressive CAA group than in the non-progressive CAA group (25 vs. 4 months, *P* < 0.001), as well as among the three groups of patients (giant CAA > medium-sized CAA > small-sized CAA, *P* < 0.001). Children with KD who had low A/G ratio were more likely to develop mid- to large-sized CAA. Nevertheless, *de novo* CAA after IVIG treatment may increase the risk of more severe arterial damage and development of progressive coronary artery damage; and both mid- to large-sized and *de novo* CAA could dramatically prolong coronary artery normalization time. Thus, aggressive risk modifications should be employed, and close monitoring with frequent echocardiography is needed for this vulnerable patient population.

## Introduction

Coronary artery aneurysm (CAA) is a potentially serious cardiac complication resulting from Kawasaki disease (KD). Moreover, it has gradually become the leading cause of acquired heart disease in children in recent years, with a case fatality rate of 0.03–1% (1–3). The administration of intravenous immunoglobulin (IVIG) together with aspirin decreases the prevalence of CAA from 25% to ~4% (2). It should also be noted that most CAA cases occur during the acute stage, and CAA regresses over time (4, 5). Furthermore, the duration and magnitude of CAAs are strongly associated with long-term prognosis (6–8). Moreover, the persistence rate of mid- to large sized CAA in acute KD phase and at 1, 3, 6, 12, and 24 months after KD onset were higher than that of small CAA. Additionally, mid- to large-sized CAA showed a slower recovery with worse prognosis (9). Similarly, Lin et al. (10) reported that the high persistence probability of mid- to large-sized CAA significantly increases the cardiovascular risk at 1 year after KD onset, when approximately two-thirds of the acute myocardial infarction cases occur. A study based on a large-scale cohort in Japan found that coronary events and major adverse cardiac events did not occur in patients with small CAA; however, they were observed in 20 (5%) and 8 (2%) of patients with medium CAA and in 82 (35%) and 45 (19%) of patients with large CAA, respectively (11). Particularly, a systematic review recently showed that mid- to large-sized CAA was one of the most significant risk factors that may reduce the survival of patients with KD (12). Furthermore, identifying the risk factors that provide early warning signs for the susceptibility of developing mid- to large-sized CAA in children with KD is clinically important, as this requires treatment with dual antiplatelet therapy (aspirin plus clopidogrel) according to the American Heart Association (AHA) guidelines (2). Meanwhile, numerous studies have shown that the changes in coronary arterial lesions toward maximal diameter beyond the acute stage may imply ongoing coronary vasculitis and dilative remodeling, resulting in worse late coronary outcomes (8, 13–19). To date, most work focused on risk factors for CAA development usually involves all types of CAAs or those classified according to their size, without making a distinction between CAA that occurred before the initial IVIG treatment and CAA that appeared *de novo* after treatment, and studies on the changes in coronary arterial lesions over time of this type of CAA are rare. The aim of the present study was to characterize CAAs, which differ in size and emergence time, determine the potential risk factors associated with CAA, and track its regression within 3 years of onset in order to provide a rationale for the prognostic evaluation value of this disease.

## Materials and methods

### Study design and population

Patients who were diagnosed with KD and received IVIG treatment at the Pediatrics Department of the First Affiliated Hospital of Guangxi Medical University, China, between January 2003 and January 2019 were recruited. The diagnostic criteria for KD were based on the guidelines proposed by the Japan Kawasaki Disease Research Committee in 2020 (20). Meanwhile, CAA was defined according to z-scores adjusted for body surface area (BSA) for a coronary artery internal diameter of ≥2.5 after 1 month of the disease course, as described in the guidelines on the diagnosis and management of cardiovascular sequelae in KD (JCS/JSCS 2020). CAA was classified based on the following z-scores: small aneurysm, 2.5 to <5; moderate aneurysm, 5 to <10; and large or giant aneurysm, ≥10 or an internal diameter of ≥8 mm (21). For patients with echocardiography follow-up, progressive CAA was defined as progressive dilatation seen across three consecutive echocardiograms, with a coronary diameter observed in the third echocardiogram 8% greater than that observed in the first one; the “8%” increase for defining progressive coronary dilatation was based on previous studies (22, 23). CAA regression was defined as the normal appearance and size (z-score of <2.5) of all coronary arteries as shown by echocardiography images, as well as normal cardiac function (2). Patients were excluded if they had recurrent KD, as the previous CAA could have persisted as a coronary complication. Furthermore, patients who did not receive IVIG treatment or received initial IVIG or corticosteroid (oral, intravenous, intramuscular, or subcutaneous) therapy at another hospital with incomplete or occasionally unavailable data required for statistical analyses were also excluded.

All patients were treated according to the AHA guidelines (2). IVIG resistance was considered when patients showed symptoms of persistent or recrudescent fever (axillary or rectal temperature ≥ 37.5 and ≥38.0°C, respectively) for at least 36 h but not longer than 7 days after receiving the initial IVIG infusion (2 g/kg) (2). All echocardiographic procedures were performed by experienced pediatric echocardiographers and re-evaluated by two additional pediatric cardiologists for confirmation. Children were classified as having CAA outcome if they had a z-score ≥ 2.5 in either the proximal right or left main coronary artery, or left anterior descending artery; moreover, only cases where the coronary artery dilation persisted for more than a month after disease onset were considered as CAA cases. Using the echocardiography reports, information on CAA status and specified CAA were registered based on the maximal z-score and diameters of all coronary arteries. All patients were first classified into three groups: non-CAA (*n* = 354), small-sized CAA (*n* = 92), medium-sized CAA (*n* = 31), and giant CAA (*n* = 7) (in order to improve statistical power, the last two groups were combined, as the giant CAA group had a small sample size). Among the 130 patients with CAA, 108 had coronary arterial lesions on admission and before the initial IVIG treatment and were placed in the CAA on-admission group. On the other hand, the remaining 22 patients developed coronary arterial lesions after the initial IVIG treatment and were placed in the CAA after-treatment group, according to the onset of ≥2.5 coronary artery z-score.

Approval for this research was required from the medical ethics committee of the First Affiliated Hospital of Guangxi Medical University [Code Number: 2021 (KY-E-240)]. Informed consent was obtained from the parents of each patient.

### Data collection

Demographic, clinical, and laboratory characteristics were obtained as follows: (1) general demographic data, including age (in months) at disease onset, sex, height, weight, and body mass index (BMI); (2) clinical characteristics, including the prevalence of incomplete KD and five principal symptoms (bilateral non-purulent conjunctivitis, perioral edema, rash, cervical lymphadenopathy of≥1.5 cm, and erythema and edema of the extremities), duration of fever before admission, illness day at treatment (considering day 1 of illness as the day when fever developed), Kobayashi score, and response to IVIG therapy; and (3) laboratory indicators, such as white blood cell count (WBC), neutrophil count, lymphocyte count, hemoglobin concentration, platelet count, aspartate aminotransferase (AST) level, alanine aminotransferase (ALT) level, total bilirubin (TSB) level, serum albumin concentration, serum sodium concentration, C-reactive protein (CRP) concentration, and erythrocyte sedimentation rate (ESR). Data on all laboratory indicators were collected for assessment during the acute febrile period and before the initial IVIG treatment. We calculated the neutrophil-to-lymphocyte count ratio (NLR), platelet-to-lymphocyte count ratio (PLR), AST-to-ALT ratio, albumin-to-globin (A/G) ratio, and CRP-to-albumin ratio based on the aforementioned indicators. Echocardiography was routinely performed in the acute stage before IVIG treatment (baseline, days < 10) and was repeated at 2 weeks (days 10–14), 1 month (days 20–40), 2 months (days 50–70), 3 months (days 80–100), and 6 months (days 170–190) after the onset of fever and then every 6 months up to a maximum of 36 months after diagnosis or until CAAs had returned to their normal size. The z-scores were calculated using Dallaire equations (https://www.pedz.de/en/welcome.html) (24) and plotted against time to evaluate how the size of the CAA evolved over time.

### Statistical analysis

The normality of distribution was verified using the Shapiro–Wilk and homogeneity tests. Data with a normal distribution were expressed as mean ± standard deviation. Two-independent sample *t*-test or one-way analysis of variance was performed to compare data between the groups. Measurement data without normal distribution were expressed as median (four-digit interval) [P_50_ (P_25_, P_75_)], and compared between groups using the Mann–Whitney U or Kruskal–Wallis H test. Enumeration data were expressed as percentages (%). The Fisher's exact test, Chi-square, or Pearson's Chi-square test was used to perform intergroup comparisons, and the Bonferroni correction was applied for multiple comparisons. Variance inflation factors were used for checking collinearity, and significant indices were analyzed using multivariate logistic regression analysis to determine risk factors. The optimum threshold for the significant parameter was constructed using receiver operating characteristic (ROC) curves. The Kaplan–Meier survival analysis was performed to compare the recovery time between cohorts using the log-rank test, and the *P*-values were two-tailed, with *P* < 0.05 considered significant. All statistical analyses were performed using IBM SPSS Statistics for Windows, version 26 (IBM Corp., Armonk, N.Y., USA).

## Results

### Baseline characteristics

A total of 484 children who were hospitalized for KD during the observation period were identified. The participants' age ranged from 2 to 158 months, and 79 (16.3%) children had primary IVIG resistance. Among them, 32 (6.6%) received corticosteroid therapy in addition to the second dose of gamma globulin therapy. Intravenous methylprednisolone was administered at a dose of 2 mg/kg per day, twice a day. Afterwards, the dose was tapered, and the administration was withdrawn until CRP concentrations normalized (<10 mg/L). Oral prednisolone administration was initiated from 2 mg/kg and reduced to 1 mg/kg and finally to 0.5 mg/kg per day, and the dose was tapered for ≥2 weeks. Almost all children were treated with aspirin, and no other biologic agents, such as infliximab, cyclosporine, anakinra, cyclophosphamide, or plasma exchange were administered during this period. Disease-related deaths were not observed in either group over a median follow-up period of 3 years.

### Comparison between the CAA groups based on z-scores for the analysis of risk factors for KD with mid- to large-sized CAA

#### Comparison of baseline characteristics

The proportion of age <1 year, fever duration before admission and days of illness at primary treatment were significantly higher in the mid- to large-sized CAA group than in the non-CAA group. However, statistically significant differences were not found between the groups in terms of sex, BMI, prevalence of incomplete KD and five principal symptoms, Kobayashi score, and IVIG resistance or corticosteroid therapy (all, *P* > 0.05). In terms of laboratory indicators, the mid- to large-sized CAA group showed significantly higher values in WBC count, platelet count, and lower A/G ratio values than the other groups. Moreover, higher proportions of serum albumin level ≤ 34 g/L were observed in the mid- to large-sized CAA group than in the non-CAA group, and higher values in neutrophil count than in the small-sized CAA group. These differences were statistically significant after Bonferroni correction (*P* < 0.016), as shown in Table 1.

**Table 1:** Demographic and clinical characteristics of the KD patients per CAA group based on z-scores.

	**Non-CAA (*n* = 354)**	**Small-sized (*n* = 92)**	**Mid- to large-sized (*n* = 38)**
**Demographic characteristics**
Age (month)	23.00 (14.00, 41.00)	18.50 (11.00, 31.00)	17.50 (7.75, 39.50)
<12 months	60 (16.9)	25 (27.2)	16 (42.1)^*^
Male	243 (68.6)	74 (80.4)	28 (73.7)
BMI (kg/m^2^)	15.43 ± 1.66	15.83 ± 1.64	15.82 ± 1.56
**Clinical characteristics**
Conjunctival injection	193 (54.5)	45 (48.9)	22 (57.9)
Changes in lips and oral cavity	192 (54.2)	42 (45.7)	20 (52.6)
Polymorphous exanthem	211 (59.6)	53 (57.6)	17 (44.7)
Cervical lymphadenopathy	170 (48.0)	42 (45.7)	16 (42.1)
Changes in extremities	113 (31.9)	28 (30.4)	13 (34.2)
Incomplete KD	89 (25.1)	20 (21.7)	9 (23.7)
Fever duration before admission (day, mean ± SD)	7.73 ± 4.25	8.70 ± 4.79	10.00 ± 5.16^*^
Days of illness at primary treatment (day)	9.55 ± 5.49	10.48 ± 5.18	12.16 ± 6.19^*^
≤ 5 days	67 (18.9)	11 (12.0)	4 (10.5)
IVIG resistance	55 (15.5)	18 (19.6)	6 (15.8)
Corticosteroid therapy	19 (5.4)	9 (9.8)	4 (10.5)
Kobayashi score (point)	1.73 ± 1.73	1.82 ± 1.65	1.84 ± 1.81
**Laboratory values**
White blood cell count ( ×10^9^/L, ref. 5–12 ×10^9^/L)	14.31 ± 6.59	14.01 ± 6.15	18.53 ± 8.21^*y^
Neutrophils count ( ×10^9^/L, ref. 1.8–6.3 ×10^9^/L)	8.84 ± 5.71	7.99 ± 5.48	11.20 ± 7.67^y^
≥80%	58 (16.4)	11 (12.0)	4 (10.5)
NLR	3.39 ± 3.73	2.98 ± 3.47	3.22 ± 3.61
Hemoglobin (g/L, ref. 120–160 g/L)	106.43 ± 13.36	105.96 ± 15.51	102.10 ± 13.71
≤ 110 g/L	210 (59.3)	56 (60.9)	26 (68.4)
Platelet count ( ×10^12^/L, ref. 125–350 ×10^12^/L)	355.85 (268.80, 450.90)	305.40 (245.25, 420.50)	466.50 (319.25, 643.35)^*y^
PLR	126.13 ± 86.56	114.12 ± 103.21	115.28 ± 66.17
CRP (mg/L, ref. 0–10 mg/L)	70.41 ± 63.05	68.72 ± 60.78	85.21 ± 53.54
Sodium (mmol/L, ref. 137–147 mmol/L)	136.56 ± 3.12	136.73 ± 3.32	136.82 ± 3.14
≤ 133 mmol/L	43 (12.1)	10 (10.9)	5 (13.2)
ALT (U/L, ref. 7–45 U/L)	50.85 ± 64.18	50.69 ± 50.83	47.79 ± 53.06
AST (U/L, ref. 13–40 U/L)	46.16 ± 55.10	54.69 ± 67.90	46.38 ± 52.15
AST/ALT ratio	1.53 ± 1.04	1.50 ± 0.90	1.39 ± 1.02
Total bilirubin (umol/L, ref. 3.4–20.5 umol/L)	8.06 ± 9.89	8.90 ± 14.11	8.01 ± 7.55
Albumin (g/L, ref. 40–55 g/L)	35.76 ± 5.54	34.92 ± 5.87	32.98 ± 5.98^*^
≤ 34 g/L	129 (36.4)	39 (42.4)	22 (57.9)^*^
A/G ratio	1.30 (1.00, 1.70)	1.30 (0.90, 1.70)	1.10 (0.81, 1.30)^*y^
CRP/albumin ratio	2.10 ± 1.99	2.14 ± 2.03	2.81 ± 2.12

Data are expressed as means with standard deviations, medians (IQR), or as number (percentage). *P*-value < 0.016 was considered statistically significant after Bonferroni correction for multiple comparisons.

^*^Statistically significant vs. non-CAA group.

^y^Statistically significant vs. small-sized CAA group.

CAA, coronary artery aneurysm; BMI, body mass index; KD, Kawasaki disease; IVIG, intravenous immunoglobulin; NLR, neutrophil-to-lymphocyte count ratio; PLR, platelet-to-lymphocyte count ratio; CRP, C-reactive protein; ALT, alanine aminotransferase; AST, aspartate aminotransferase; AST/ALT, aspartate aminotransferase-to-alanine aminotransferase; A/G, albumin-to-globin.

#### Analysis of risk factors for KD with mid- to large-sized CAA

**Figure 1:**
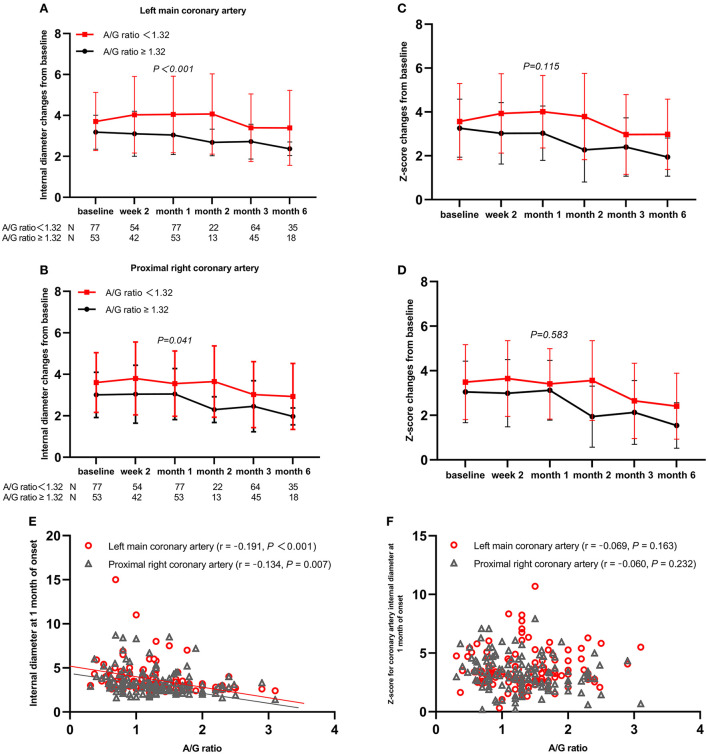
Coronary artery changes from baseline values over time by subgroups and Spearman's correlation analysis between A/G ratio and coronary artery outcomes. **(A)** Left main coronary artery; **(B)** Proximal right coronary artery; **(C)** z-score of left main coronary artery; **(D)** z-score of proximal right coronary artery; **(E)** coronary artery internal diameter; **(F)** z-scores of coronary artery. A/G, albumin-to-globin.

Mid-to-large-sized CAA was significantly associated with the five baseline laboratory variables (WBC count, neutrophil count, platelet count, proportions of serum albumin level ≤ 34 g/L, and A/G ratio) and three demographic and clinical variables (proportions of age < 1 year, fever duration before admission, and days of illness at primary treatment). The multivariable analysis included WBC count instead of neutrophil count because %neutrophils, which was not different among the three groups, as well as WBC (25), is reportedly a risk factor of CAA development. All variables were tested for collinearity; however, no collinearity was present. The A/G ratio was an independent risk factor for mid- to large-sized CAA (Table 2), and the area under the ROC curve for the value of the A/G ratio was 0.637 (95% confidence interval: 0.551–0.724). As with the optimal cutoff value of 1.32, the sensitivity and specificity of the A/G ratio for predicting mid- to large-sized CAA in patients with KD were 79 and 49%, respectively. From baseline to 2 and 4 weeks and later 3 months, patients with an A/G ratio of <1.32 had larger coronary artery internal diameter, and the differences between the two groups were statistically significant at 1 month after onset (Figures 1A,B). However, the differences in the z-scores of coronary arteries were not significantly different (Figures 1C,D). In addition, at 1 month of the disease course, the A/G ratio negatively correlated with the internal diameter of the left main coronary artery (*r* = −0.191, *P* < 0.001) and proximal right coronary artery (*r* = −0.134, *P* = 0.007) (Figure 1E). However, correlation was not observed between the A/G ratio and z-scores of coronary arteries (*r* = −0.069, *P* = 0.163 for the left main coronary artery and *r* = −0.060, *P* = 0.232 for the proximal right coronary artery) (Figure 1F).

**Table 2:** Relationship between risk factors and the formation of mid-to-large sized CAA, compared to other CAA groups.

	**Univariate**				**Multivariate**				**VIF**
	**Non-CAA**		**Small-sized CAA**		**Non-CAA**		**Small-sized CAA**		
	**Odds ratio (95%CI)**	***P*-value**	**Odds ratio (95%CI)**	***P*-value**	**Odds ratio (95%CI)**	***P*-value**	**Odds ratio (95%CI)**	***P*-value**	
Age <1 year (reference: age ≥ 1 year)	0.281 (0.139–0.566)	<0.001	0.513 (0.233–1.132)	0.098	0.217 (0.096–0.494)	<0.001	0.438 (0.175–1.095)	0.077	1.123
Fever duration before admission	0.909 (0.853–0.968)	0.003	0.954 (0.889–1.024)	0.189	0.977 (0.864–1.105)	0.710	1.045 (0.911–1.199)	0.527	3.514
Days of illness at primary treatment	0.931 (0.885–0.980)	0.006	0.960 (0.906–1.017)	0.166	0.945 (0.856–1.043)	0.260	0.938 (0.838–1.049)	0.262	3.178
White blood cell count	0.926 (0.887–0.967)	<0.001	0.919 (0.873–0.968)	0.001	0.949 (0.904–0.997)	0.038	0.955 (0.902–1.010)	0.109	1.210
Platelet count	0.997 (0.995–0.999)	<0.001	0.996 (0.993–0.998)	<0.001	0.999 (0.997–1.001)	0.376	0.997 (0.995–0.999)	0.013	1.290
Albumin ≤ 34 g/L (reference: albumin > 34 g/L)	0.417 (0.211–0.823)	0.012	0.535 (0.249–1.150)	0.109	0.735 (0.297–1.818)	0.505	1.077 (0.394–2.944)	0.885	1.498
A/G ratio	3.027 (1.401–6.540)	0.005	3.228 (1.393–7.482)	0.006	3.802 (1.237–11.689)	0.020	4.123 (1.245–13.659)	0.020	1.756

CAA, coronary artery aneurysm; CI, confidence interval; VIF, variance inflation factors; A/G, albumin-to-globin.

### Comparison between the CAA groups based on the onset of z-score for the analysis of risk factors for *de novo* CAA after IVIG treatment

#### Comparison of baseline characteristics

Fever duration before admission and days of illness at primary treatment were significantly lower in the CAA after-treatment group (*P* < 0.05). Regarding the clinical outcomes, the CAA after-treatment group needed a longer time to reach the peak of CAA diameters and showed a higher incidence of progressive CAA than the CAA on-admission group, with statistically significant differences (*P* < 0.05). However, statistically significant differences were not found between the groups regarding demographic characteristics and laboratory indicators (all, *P* > 0.05), as shown in Table 3.

**Table 3:** Demographic and clinical characteristics of the patients with CAA between groups based on the emergence time of coronary artery aneurysm.

	**Total (*n* = 130)**	**CAA after-treatment (*n* = 22)**	**CAA on-admission (*n* = 108)**	***P*-value**
**Demographic characteristics**
Age (month)	25.45 ± 24.83	28.14 ± 18.55	24.90 ± 25.97	0.579
<1 year	41 (31.5)	4 (18.2)	37 (34.3)	0.139
Male	102 (78.5)	16 (72.7)	86 (79.6)	0.665
BMI (kg/m^2^)	15.83 ± 1.61	15.33 ± 1.29	15.93 ± 1.66	0.109
**Clinical characteristics**
Conjunctival injection	67 (51.5)	15 (68.2)	52 (48.1)	0.087
Changes in lips and oral cavity	62 (47.7)	14 (63.6)	48 (44.4)	0.100
Polymorphous exanthem	70 (53.8)	16 (72.7)	54 (50.0)	0.051
Cervical lymphadenopathy	58 (44.6)	13 (59.1)	45 (41.7)	0.134
Changes in extremities	41 (31.5)	9 (40.9)	32 (29.6)	0.299
Incomplete KD	29 (22.3)	6 (27.3)	23 (21.3)	0.739
Fever duration before admission (day)	8.00 (5.00, 12.00)	7.00 (4.00, 8.00)	9.00 (5.00, 12.75)	0.023
Days of illness at primary treatment (day)	10.00 (7.00, 14.25)	8.00 (6.00, 11.25)	10.00 (7.00, 15.00)	0.038
≤ 5 days	15 (11.5)	4 (18.2)	11 (10.2)	0.481
IVIG resistance	24 (18.5)	4 (18.2)	20 (18.5)	1.000
Corticosteroid therapy	13 (10.0)	1 (4.5)	12 (11.1)	0.585
Kobayashi score (point)	1.82 ± 1.69	1.64 ± 2.24	1.86 ± 1.57	0.572
**Laboratory values**
White blood cell count ( ×10^9^/L, ref. 5–12 ×10^9^/L)	15.33 ± 7.09	14.72 ± 5.35	15.46 ± 7.41	0.659
Neutrophils count ( ×10^9^/L, ref. 1.8–6.3 ×10^9^/L)	8.93 ± 6.34	9.61 ± 4.86	8.79 ± 6.61	0.580
≥80%	15 (11.5)	3 (13.6)	12 (11.1)	1.000
NLR	3.05 ± 3.50	4.13 ± 4.16	2.82 ± 3.33	0.111
Hemoglobin (g/L, ref. 120–160 g/L)	104.83 ± 15.05	104.50 ± 14.58	104.90 ± 15.22	0.910
≤ 110 g/L	82 (63.1)	14 (63.6)	68 (63.0)	0.952
Platelet count ( ×10^12^/L, ref. 125–350 ×10^12^/L)	387.22 ± 210.20	368.95 ± 158.58	390.94 ± 219.66	0.656
PLR	114.46 ± 93.65	120.01 ± 67.41	113.33 ± 98.35	0.762
CRP (mg/L, ref. 0–10 mg/L)	73.54 ± 59.03	86.90 ± 68.67	70.81 ± 56.84	0.245
ESR (mm/H, ref. 0–20 mm/H)	58.32 ± 31.88	63.24 ± 30.17	57.32 ± 32.26	0.429
Sodium (mmol/L, ref. 137–147 mmol/L)	136.75 ± 3.26	135.82 ± 3.72	136.95 ± 3.14	0.139
ALT (U/L, ref. 7–45 U/L)	49.84 ± 51.30	63.56 ± 66.49	47.05 ± 47.53	0.170
AST (U/L, ref. 13–40 U/L)	52.26 ± 63.61	60.83 ± 97.96	50.51 ± 54.57	0.490
AST/ALT ratio	1.47 ± 0.93	1.22 ± 0.79	1.52 ± 0.95	0.163
Total bilirubin (umol/L, ref. 3.4–20.5 umol/L)	5.55 (3.50, 9.20)	7.00 (3.85, 12.83)	5.30 (3.50, 9.08)	0.260
Albumin (g/L, ref. 40–55 g/L)	34.35 ± 5.94	33.71 ± 5.09	34.48 ± 6.11	0.581
≤ 34 g/L	61 (46.9)	10 (45.5)	51 (47.2)	0.880
A/G ratio	1.31 ± 0.56	1.26 ± 0.45	1.33 ± 0.58	0.597
CRP/albumin ratio	2.34 ± 2.07	2.71 ± 2.22	2.26 ± 2.04	0.352
**Clinical outcomes**
Time to reach the peak of CAA diameters (month)	0.50 (0.25, 1.00)	1.00 (0.50, 1.25)	0.50 (0.25, 1.00)	<0.001
Mid-to large-sized CAA	38 (29.2)	3 (13.6)	35 (32.4)	0.078
Progressive CAA	44 (33.8)	13 (59.1)	31 (28.7)	0.006

Data are expressed as means with standard deviations, medians (IQR), or as number (percentage). CAA, coronary artery aneurysm; BMI, body mass index; KD, Kawasaki disease; IVIG, intravenous immunoglobulin; NLR, neutrophil-to-lymphocyte count ratio; PLR, platelet-to-lymphocyte count ratio; CRP, C-reactive protein; ESR, erythrocyte sedimentation rate; ALT, alanine aminotransferase; AST, aspartate aminotransferase; AST/ALT, aspartate aminotransferase-to-alanine aminotransferase; A/G, albumin-to-globin.

#### Comparison of time-course changes of coronary artery z-scores

**Figure 2:**
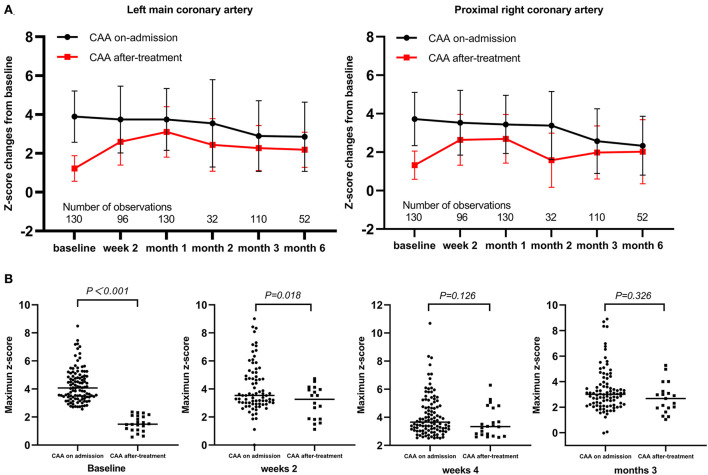
Time-course changes of coronary artery z-scores and maximum values. **(A)** Change of z-score at each coronary artery segment as mean (SD); **(B)** Maximum z-scores. CAA, coronary artery aneurysm.

**Figure 3:**
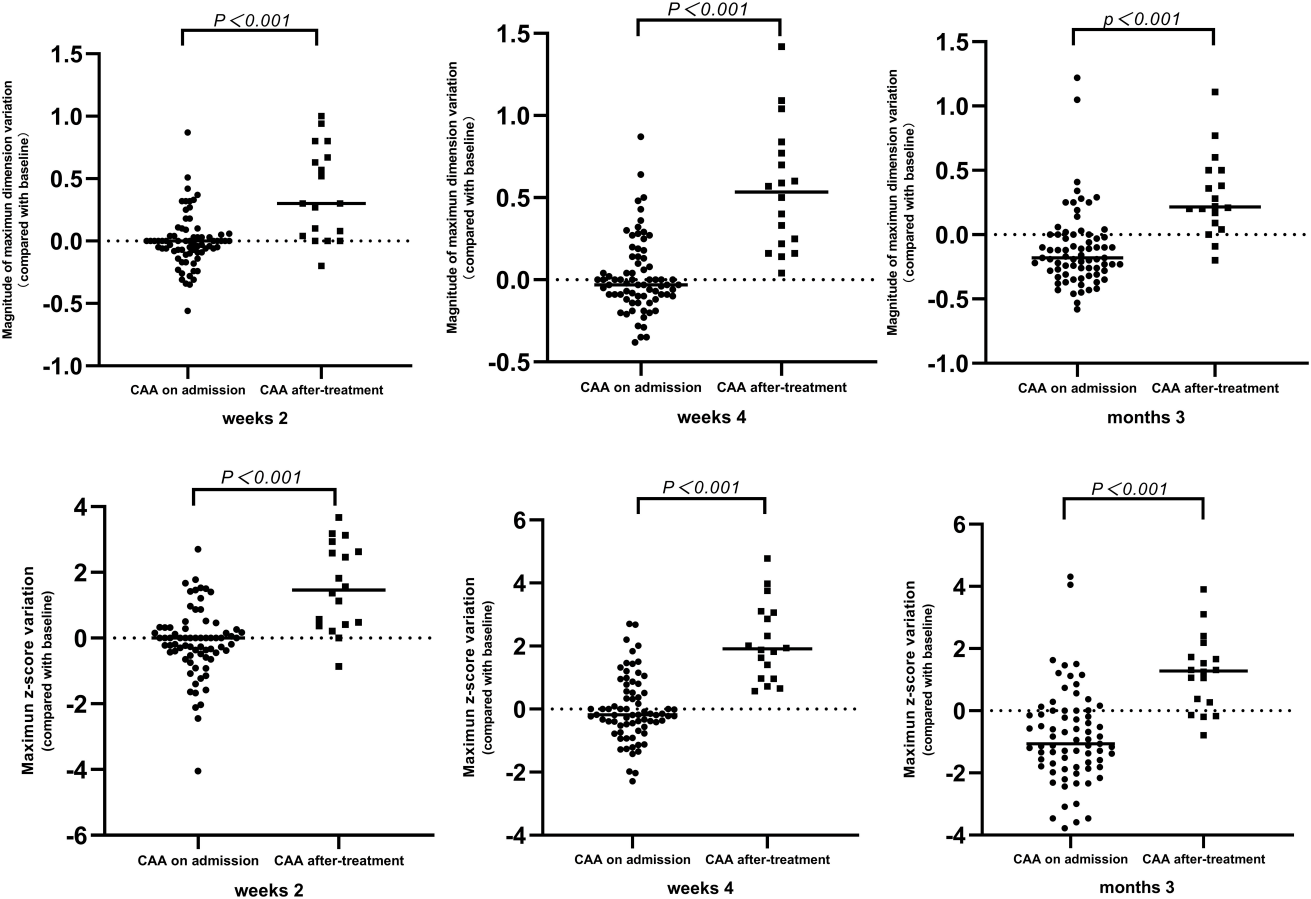
Time-course changes of magnitude of CAA dimension variation and mean difference of maximum z-score. CAA, coronary artery aneurysm.

Among the 130 patients with echocardiography follow-up, a change of z-score at each coronary artery segment was observed in patients with *de novo* CAA after IVIG treatment. Moreover, the coronary artery showed an increasing trend in z-scores from 2 to 4 weeks (Figure 2A). The maximum z-score was significantly higher in patients with CAA on admission within 2 weeks of illness onset; however, significant differences were not observed between the two groups after 2 weeks (Figure 2B). A total of 91 patients were observed whose data for coronary outcomes were complete from baseline to 2 and 4 weeks and later 3 months (Supplementary Table 1), and their coronary artery changes were compared with baseline values; patients with *de novo* CAA after IVIG treatment had significantly greater changes in both the magnitude of CAA dimension variation and the maximum z-score of the coronary artery at 2 and 4 weeks and then 3 months after onset (*P* < 0.001) (Figure 3).

### Long-term outcomes

**Figure 4:**
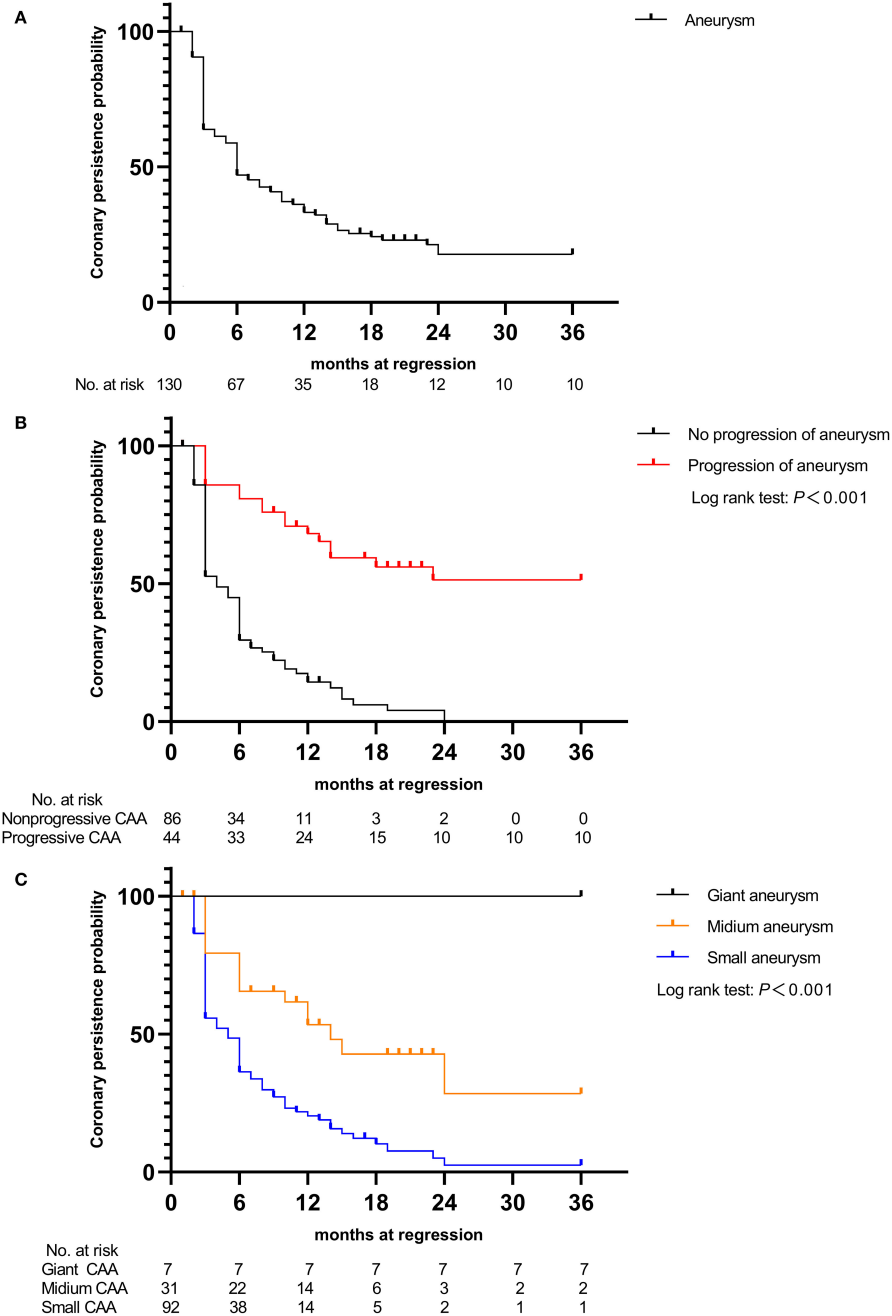
Kaplan–Meier event-free curves showing the probability with persistent coronary artery aneurysms **(A)** in those with coronary aneurysms, **(B)** in patients with and without progressive aneurysms, and **(C)** in the three groups of patients (small, medium, and giant coronary aneurysm groups). CAA, coronary artery aneurysm.

**Figure 5:**
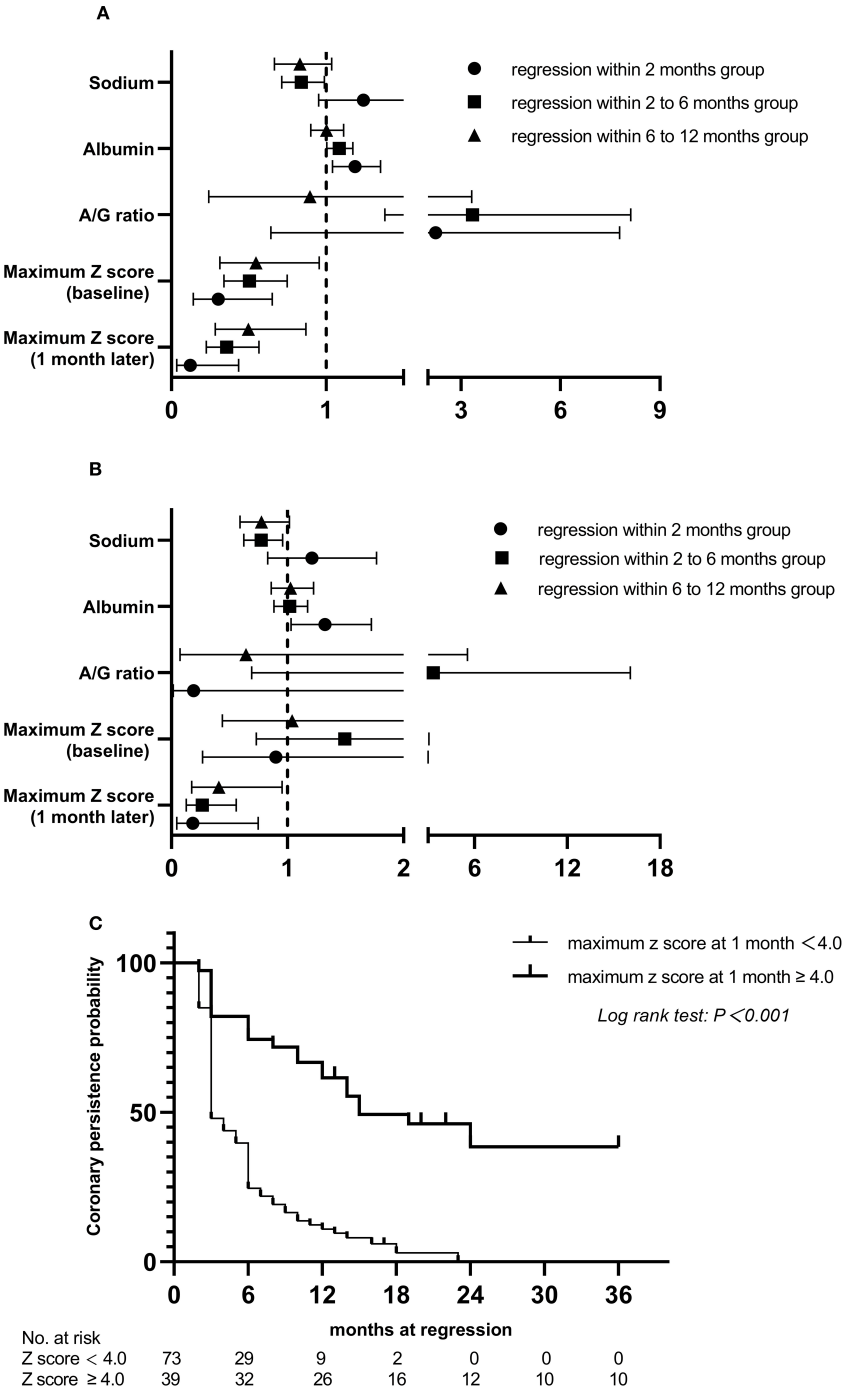
Relationship between risk factors and the persistence time of coronary artery aneurysm, compared to persistent at >1 year group and Kaplan. Meier event-free curves showing the probability with persistent coronary artery aneurysms within 3 years by subgroups. **(A)** univariate analysis; **(B)** multivariate analysis; **(C)** in patients with and without maximum z score at 1 month ≥ 4.0. A/G, albumin-to-globin.

At the end of the 3-year follow-up period, acute myocardial infarction (AMI) did not occur in any patient. The median intervals for giant CAAs to reach their greatest size (8.2–15.0 mm) was 4.5 months, ranging from 1.5 to 12.0 months; for medium-sized CAAs (4.3–7.2 mm) was 1.0 month, ranging from 0.5 to 3.0 months; and for small-sized CAAs (2.5–5.1 mm) was 0.5 month, ranging from 0.25 to 1.0 month. Patients with mid- to large-sized CAA had significantly longer progressive coronary diameter dilatation than those with small-sized CAA (1.7 vs. 1.0 months, *P* = 0.004). Over the 3-year follow-up period, 9.2% of patients [12/130 (three medium-sized and nine small-sized CAAs)] were lost to follow-up within 6 months, 5.1% [6/118 (three medium-sized and three small-sized CAAs)] within 6–12 months, 8.9% [10/112 (seven medium-sized and three small-sized CAAs)] within 1–2 years, and none after 2 years. Coronary aneurysms persisted in 9.8% (10/102) of the patients during follow-up (Figure 4A). Moreover, 44 (33.8%) patients had progressive CAA, [seven (5.3%) giant CAA, 16 (12.3%) medium-sized CAA, and 21 (16.2%) small-sized CAA]. Kaplan–Meier survival analysis revealed that the estimated median time of aneurysm persistence was significantly higher in the progressive CAA group than in the non-progressive CAA group (25 vs. 4 months, *P* < 0.001) (Figure 4B). All giant aneurysms persisted during follow-up. The probability of coronary aneurysm persistence at 6 months and at 1, 2, and 3 years after KD onset for medium-sized aneurysms was 78.6% (22/28), 56.0% (14/25), 16.7% (3/18), and 11.1% (2/18), respectively, while that for small-sized aneurysms was 45.8% (38/83), 17.5% (14/80), 2.6% (2/77), and 1.3% (1/77), respectively (Figure 4C). Of the 112 patients followed for ≥1 year, 16.1% (18/112) developed coronary artery lesions after the initial IVIG treatment, of which 22.2% (4/18) had persisted until the 1-year follow-up period. Moreover, 70.5% of patients (79/112) experienced CAA regression within 1 year of acute illness [12 (10.7%) within 2 months, 53 (47.3%) within 2–6 months, and 14 (12.3%)] within 6–12 months); the baseline characteristics and results of the multivariate analysis of risk factors associated with regression of coronary aneurysms are presented in the Supplementary material. The maximum z-score at 1 month was a significant independent predictor of CAA persistence at > 1 year after adjusting for age and sex (Figures 5A,B). Kaplan–Meier survival analysis revealed that the estimated median time of aneurysm persistence was significantly higher in patients with a maximum z-score ≥ 4 at 1 month than in those with a maximum z-score < 4 (15 vs. 3 months, *P* < 0.001) (Figure 5C).

## Discussion

The potential risk factors and coronary artery outcomes associated with CAAs, which differ in size and emergence time, were evaluated in this study. We observed that a lower A/G ratio was highly associated with mid- to large-sized CAA development and negatively correlated with the absolute internal diameter of the coronary artery at 1 month after onset. Moreover, a significantly longer time to reach the peak CAA diameter, higher incidence of progressive CAA, and greater changes in both the magnitude of CAA dimension variation and maximum z-score of the coronary artery at 2 and 4 weeks and then 3 months after onset were observed among patients with KD and *de novo* CAA after IVIG treatment than in those with CAA on admission. Moreover, these factors may significantly prolong the time required to achieve coronary artery normalization in these patients.

The development of CAA is the most severe complication of KD. Other complications such as cardiac sequelae caused by mid- to large-sized CAA are associated with severe morbidity in pediatric patients. The Kobayashi score is now widely used in Japanese populations to stratify the risk for IVIG resistance and reduce adverse coronary artery outcomes by adding adjunctive anti-inflammatory medications to the primary IVIG treatment (26, 27). Unfortunately, the Kobayashi score did not accurately predict poor outcomes in non-Japanese populations, as was also identified in the present study. Moreover, the intensive treatment regimen in high-risk KD is controversial (28, 29). Thus, the patients in our population were not administered any steroids or biologic agents concomitantly with the initial IVIG treatment. In this study, the CAA incidence was 26.9%, where the incidence of mid- to large-sized CAA was 7.9%, which was higher than that reported in the literature (30). This may be because many previous studies used the criteria of the Japanese Ministry of Health for coronary abnormalities, which may underdiagnose the severity of CAA. In addition, a substantial number of patients with severe KD or those with failed medical treatment were referred to our hospital. In the current study, we found a strong statistically significant trend of decreasing serum albumin concentrations across patient categories (non > small-sized > mid- to large-sized). Furthermore, the A/G ratio negatively correlated with the internal diameter of the coronary artery at 1 month of onset and was associated with mid- to large-sized CAA development. These are in line with the results of a previous study on hepatic dysfunction secondary to KD, indicating that the A/G ratio serves as an independent predictor of CAA at a cutoff value of <1.48 (31). Similarly, our results revealed that a lower A/G ratio indicates a greater risk for CAA development, as our study showed that a larger CAA size indicates a lower A/G ratio. Moreover, the risk of KD complicated with mid- to large-sized CAA increases when A/G ratio <1.32. Notably, further analysis revealed that the coronary artery internal diameter of patients with an A/G ratio of <1.32 was larger than that of those with an A/G ratio of ≥1.32 within 3 months of disease onset. Therefore, a future longitudinal study on KD should delineate the effect of the A/G ratio on the dynamic changes of the internal diameter of the coronary artery, as the early detection of risk factors can be beneficial.

Based on echocardiographic data, 20–30% of patients with KD will develop transient coronary artery dilatation, and 5–9% will develop coronary artery aneurysms despite timely IVIG therapy (32, 33). IVIG has long been recognized as the first-line medication for acute KD; however, its specific mechanisms have not been thoroughly elucidated. Histopathological changes in the coronary media of patients with KD occur as early as days 6–8 of the disease course (34). Moreover, IVIG administration inhibits the inflammatory response and neutralizes inflammatory factors, as early termination of systemic inflammation is essential to prevent vasculitis progression (35–37). Considering this, we speculate that patients with *de novo* CAA after IVIG treatment may have a more severe systemic inflammatory response, which induces subsequent worsening of coronary artery damage, which was also confirmed in this study. Our study showed that patients with *de novo* CAA after IVIG treatment had shorter fever duration prior to admission and earlier initiation of therapy. However, the z-scores of the coronary arteries showed an increasing trend after the end of IVIG infusion, although significant differences were not observed between the groups regarding the incidence of IVIG resistance and proportion of days of illness at primary treatment of ≤5 days, which were previously reported as risk factors for CAA development (38). On the other hand, higher proportions of the five principal symptoms, higher levels of both CRP and ESR, higher values of both NLR and CRP/albumin ratio, and lower serum sodium and albumin concentrations were observed in the CAA after-treatment group than in the CAA on-admission group. Although these differences were not statistically significant given the small sample size, their magnitude was meaningful and suggested that a strong inflammatory response stimulates the increased permeability of the endothelium (11, 39, 40), thereby further supporting our speculation. Further analysis is needed to clarify these associations, as this information has great clinical significance.

Persistent low-grade inflammation may be associated with progressive coronary dilatation, and a progressive increase in CAA size was strongly correlated with increased incidence of major adverse cardiovascular events, as shown in two previous studies (22, 41). Our study found that patients with mid- to large-sized CAA were more likely to have longer progressive dilatation of coronary arteries than those with small-sized CAA and to show a slower recovery, which is in line with the results of previous studies (10, 22). Moreover, the coronary arterial internal diameters tended to ascend within 2–4 weeks of onset in patients with *de novo* CAA after IVIG treatment, of which 59.1% (13 of 22) developed progressive CAA. Patients with a greater change in z-score or the magnitude of CAA dimension variation during KD have been associated with risk factors for subsequent coronary involvement (41, 42). Hence, the significantly greater changes in both the magnitude of CAA dimension variation and maximum z-score of the coronary artery at 2 and 4 weeks and then 3 months after onset that were observed in patients with *de novo* CAA after IVIG therapy in this study. Furthermore, in 22.2% (4/18) of patients with *de novo* CAA after IVIG treatment, the CAA persisted until the 1-year follow-up examination (see Supplementary material). These findings suggest that *de novo* CAA after IVIG treatment may add to the risk for developing more severe arterial damage from KD. To the best of our knowledge, this is the first report on the features and coronary artery outcomes of patients with KD stratified based on the emergence time of CAA. Our observations suggest that continuous attention should be paid to these patients and that the subgroup of patients with KD and *de novo* CAA after IVIG treatment needs more frequent follow-up.

Previous studies have demonstrated the ongoing changes in coronary morphology in patients with KD with coronary artery lesions after the acute phase, especially within 1 year of onset (18, 19). Small CAAs often regressed at 2 months, and most CAAs sized <6 mm regress by approximately 6 months after KD (43, 44). Friedman et al. reported that CAA regression occurred in 75% of cases over a 2-year period in a United States population (45), and a large Japanese study showed that regression occurs in 55–60% of CAAs, typically within 1–2 years of acute illness (13), which was in accordance with our finding of 70.5%. Additionally, subgroup analyses found that the maximum z-score at 1 month was significantly negatively associated with CAA regression within 1 year, and patients with a maximum z-score at 1 month ≥ 4 showed a significantly slower recovery over the 3-year follow-up period (Supplementary material). This further confirms the results of earlier studies showing that aneurysm severity at 1 month after KD onset was the only independent risk factor for aneurysm persistence (10, 45). To date, the AHA recommends dual antiplatelet therapy for prophylaxis against thrombosis only for patients with intermediate to large CAAs (2). However, studies have found that the incidence rate of CAA stenosis or occlusion was 46–61% in Japan (46), 42.3% in South Korea (47), and 34.6% in the United States (48) in patients on monotherapy with low dose aspirin, suggesting that aspirin alone is not enough to prevent CAA thrombosis, even in those with small CAAs. This study provides evidence of the potential value of risk stratification among patients with early-stage CAA, which may help identify high-risk patients for targeted intervention, which can in turn effectively inhibit inflammation and subsequent coronary arterial remodeling. Future randomized controlled trials are certainly warranted to definitively confirm our findings.

Our study has several limitations. First, it was a single-center retrospective study with a long observation period, and thus, it was susceptible to selection bias. The diagnosis and timely treatment of KD may have improved over time, but recent practice recommendations still follow the criteria published in 1993 and 1994 (49, 50). Thus, to minimize selection bias, we strictly applied the most up-to-date recommendations and inclusion and exclusion criteria to further verify and select the target population. Second, the small sample size of the subgroup of patients with KD and *de novo* CAA after IVIG treatment limited the statistical power. Moreover, missing data on the left anterior descending artery with small-sized CAA were not analyzed because dilatation was not detected. Furthermore, the numbers of patients with medium and giant CAAs were 31 and 7, respectively, and only patients with giant CAA or thrombosis were examined by coronary angiography, since coronary angiography is an invasive examination. Therefore, comparison between echocardiography results and coronary angiography findings could not be made, and some indicators that are associated with CAA recovery were not available in the study, such as the configuration and number of CAAs (12, 51–53). Hence, these findings should be interpreted with caution, and large-scale and more in-depth studies are needed to confirm our findings. Third, the coronary artery z-score was not based on the normal value for Chinese children. However, z-scores calculated by Dallaire equations to define CAA have been generally used at our hospital since 2011. Therefore, we re-evaluated all echocardiographs and calculated the z-scores based on this equation for consistency. Fourth, the regression of CAA as detected through echocardiography might show earlier than the checked time point. Additionally, a small number of patients were lost to follow-up, and a 3-year follow-up period may not have been sufficient to determine the outcomes of all patients with CAA. Therefore, a multicenter study with a large cohort and long-term longitudinal follow-up is required.

## Conclusions

Children with KD who had low A/G ratio were more likely to develop mid- to large-sized CAA. Nevertheless, *de novo* CAA after IVIG treatment may increase the risk of more severe arterial damage and development of progressive coronary artery damage, and both mid- to large-sized and *de novo* CAA could dramatically prolong coronary artery normalization time. Thus, aggressive risk modifications should be employed, and close monitoring with frequent echocardiography is needed for this vulnerable patient population.

## Data availability statement

The original contributions presented in the study are included in the article/Supplementary material, further inquiries can be directed to the corresponding author.

## Ethics statement

Written informed consent was obtained from the individual(s), and minor(s)' legal guardian/next of kin, for the publication of any potentially identifiable images or data included in this article.

## Author contributions

JL drafted the manuscript, contributed to the data collection, performed the statistical analysis, and approved the final manuscript as submitted. QY provided the figures, contributed to the data collection, study design, and approved the final manuscript as submitted. SQ contributed to the data collection and approved the final manuscript as submitted. DS administered primary treatment to these patients while they were admitted and contributed to the study design, and approved the final manuscript as submitted. BY prepared the tables, contributed to the data collection, and approved the final manuscript as submitted. YP conceived and designed the study, contributed to the data collection, and approved the final manuscript as submitted. All authors contributed to the article and approved the submitted version.

## Funding

Guangxi Medical and health key discipline construction project 2019 (19), and Guangxi Clinical Research Center for Pediatric Disease (no.: GUI KE AD22035219).

## Conflict of interest

The authors declare that the research was conducted in the absence of any commercial or financial relationships that could be construed as a potential conflict of interest.

## Publisher's note

All claims expressed in this article are solely those of the authors and do not necessarily represent those of their affiliated organizations, or those of the publisher, the editors and the reviewers. Any product that may be evaluated in this article, or claim that may be made by its manufacturer, is not guaranteed or endorsed by the publisher.

## Abbreviations

AHA: American Heart Association
ALT: alanine aminotransferase
AMI: Acute myocardial infarction
AST: aspartate aminotransferase
A/G: albumin to globin
BMI: body mass index
BSA: body surface area
CAA: Coronary artery aneurysm
CRP: C-reactive protein
CI: confidence interval
ESR: erythrocyte sedimentation rate
IVIG: intravenous immunoglobulin
KD: Kawasaki Disease
NLR: neutrophil-to-lymphocyte count ratio
PLR: platelet-to-lymphocyte count ratio
ROC: receiver operating characteristic
TSB: total serum bilirubin
WBC: white blood cell count.
