# Supplementary material for: Risk factors and coronary artery outcomes of coronary artery aneurysms differing in size and emergence time in children with Kawasaki disease
Source: Front Cardiovasc Med. 2022 Sep 9;9:969495. doi: 10.3389/fcvm.2022.969495 (PMC9505689; doi:10.3389/fcvm.2022.969495)
Supplement: Supplementary file 1 [file Table_1.DOCX]

Supplementary table 1

Coronary artery dimension and z-score summaries according to the emergence time of coronary artery aneurysm

|  | Total (n=91) | CAA after-treatment (n=18) | CAA on-admission (n=73) | *P-value* |
| --- | --- | --- | --- | --- |
| Left main coronary artery | | | | |
| Baseline [*P*_50_ (*P*_25_, *P*_75_)] | 3.20 (2.80, 4.00) | 2.30 (2.00, 2.50) | 3.40 (2.95, 4.15) | < 0.001 |
| Week 2 [mean±SD] | 3.61±1.67 | 3.01±0.78 | 3.76±1.80 | 0.088 |
| Month 1 [mean±SD] | 3.79±1.87 | 3.38±0.99 | 3.89±2.02 | 0.300 |
| Month 3 [*P*_50_ (*P*_25_, *P*_75_)] | 2.70 (2.40, 3.40) | 2.65 (2.40, 3.23) | 2.70 (2.50, 3.50) | 0.418 |
| Proximal right coronary artery | | | | |
| Baseline [*P*_50_ (*P*_25_, *P*_75_)] | 2.80 (2.20, 4.00) | 2.00 (1.70, 2.20) | 3.20 (2.60, 4.30) | < 0.001 |
| Week 2 [mean±SD] | 3.43±1.67 | 2.89±1.03 | 3.56±1.77 | 0.127 |
| Month 1 [*P*_50_ (*P*_25_, *P*_75_)] | 2.90 (2.40, 3.90) | 2.65 (2.40, 3.05) | 3.00 (2.35, 4.00) | 0.268 |
| Month 3 [mean±SD] | 2.80±1.51 | 2.52±1.07 | 2.87±1.60 | 0.388 |
| magnitude of maximun dimension variation (compared with baseline)^a^ | | | | |
| Week 2 [*P*_50_ (*P*_25_, *P*_75_)] | 0.00 (-0.07, 0.18) | 0.30 (0.03, 0.70) | 0.00 (-0.09, 0.05) | < 0.001 |
| Month 1 [*P*_50_ (*P*_25_, *P*_75_)] | 0.00 (-0.09, 0.27) | 0.53 (0.20, 0.79) | -0.03 (-0.10, 0.13) | < 0.001 |
| Month 3 [mean±SD] | -0.03±0.34 | 0.30±0.32 | -0.12±0.30 | < 0.001 |
| Maximun z-score variation (compared with baseline)^b^ | | | | |
| Week 2 [*P*_50_ (*P*_25_, *P*_75_)] | 0.00 (-0.40, 0.87) | 1.47 (0.40, 2.71) | 0.00 (-0.51, 0.31) | < 0.001 |
| Month 1 [mean±SD] | 0.42±1.36 | 2.13±1.23 | 0.00±1.03 | < 0.001 |
| Month 3 [mean±SD] | -0.43±1.66 | 1.21±1.22 | -0.83±1.50 | < 0.001 |

CAA, coronary artery aneurysm.

^a^ The magnitude of change of difference between the maximun internal diameter of coronary artery at 2 and 4 weeks and the 3 months after onset and the maximun internal diameter of coronary artery at baseline = (absolute internal diameter of coronary artery at 2 and 4 weeks and then 3 months after onset) − (absolute internal diameter of coronary artery at baseline)/absolute internal diameter of coronary artery at baseline;

^b^ Difference between the maximun z-score of coronary artery at 2 and 4 weeks and then 3 months after onset and the maximun z-score of coronary artery at baseline = (z-score of coronary artery at 2 and 4 weeks and then 3 months after onset) − (z-score of coronary artery at baseline).
