# Supplementary material for: Risk factors and coronary artery outcomes of coronary artery aneurysms differing in size and emergence time in children with Kawasaki disease
Source: Front Cardiovasc Med. 2022 Sep 9;9:969495. doi: 10.3389/fcvm.2022.969495 (PMC9505689; doi:10.3389/fcvm.2022.969495)
Supplement: Supplementary file 2 [file Data_Sheet_1.docx]

Supplementary File

Of the 130 included patients, 112 have completed 1-year follow-up, including 7 giant coronary artery aneurysm (CAA), 25 medium-sized CAA and 80 small-sized CAA. Of the 112 patients followed for ≥1 year, 16.1% (18/112) developed coronary artery lesions after the initial IVIG treatment, of which 22.2% (4/18) had persisted until the 1-year follow-up period. Moreover, 70.5% of patients (79/112) experienced CAA regression within 1 year of acute illness. Patients were divided into four groups according to the regression time: within 2 months group (n=12), within 2 to 6 months group (n=53), within 6 to 12 months group (n=14) and persistent at > 1 year group (n=33), and the association between baseline characteristics with CAA regression was investigated among the groups. Differences in serum sodium and albumin concentrations, albumin-to-globin (A/G) ratio, and maximum z-score at baseline and 1 months of onset between groups were statistically significant. In terms of clinical outcomes, the persistent at > 1 year group showed significant higher proportion of mid- to large-sized CAA and incidence of thrombosis than the other groups. However, statistically significant differences were not found between the groups in terms of demographic and clinical characteristics (all, *P* > 0.05), as shown in Table 1. To determine the relative effect of each risk factor for KD patients with persistent CAA, we performed a logistic regression analysis. Serum levels of sodium and albumin, A/G ratio, and maximum z-score at baseline and 1 months were included in the multivariable model. All variables were tested for collinearity; however, no collinearity was present. Ultimately, the maximum z-score at 1 months of onset was an independent risk factor for CAA persistence at > 1 year after adjusting for age and sex (Table 2), and the area under the receiver operating characteristic curve for the value of the maximum z-score at 1 month was 0.809 (95% confidence interval: 0.714–0.904). As with the optimal cutoff value of 4.0, the sensitivity and specificity of the maximum z-score at 1 month for predicting CAA persistent at > 1 year in patients with KD were 73% and 82%, respectively (Figure 1).

Figure 1 Receiver operating characteristic curve and AUC for CAA persistence at > 1 year in patients with KD

AUC, area under curve; CAA, coronary artery aneurysm; KD, Kawasaki disease.

Table 1 Demographic and clinical characteristics of the Kawasaki disease patients per CAA-group based on regression time

|  | within 2 months  (n=12) | 2-6 months  (n=53) | 6-12 months  (n=14) | persistent at > 1 year group  (n=33) |
| --- | --- | --- | --- | --- |
| **Demographic characteristics** | | | | |
| Age [month] | 28.33±9.96 | 26.62±25.24 | 23.79±12.50 | 27.12±31.22 |
| < 12 months | 0 | 19 (35.8) | 2 (14.3) | 12 (36.4) |
| Male | 10 (83.3) | 40 (75.5) | 8 (57.1) | 27 (81.8) |
| BMI [kg/m^2^] | 15.23±0.80 | 15.73±1.65 | 15.90±1.28 | 16.12±1.97 |
| **Clinical characteristics** | | | | |
| Incomplete KD | 2 (16.7) | 9 (17.0) | 0 | 6 (18.2) |
| Fever duration before admission  [day] | 8.00±4.29 | 8.06±3.98 | 9.21±4.93 | 10.52±5.43 |
| Days of illness at primary treatment [day] | 10.25±4.62 | 10.34±5.94 | 10.71±5.36 | 12.00±5.53 |
| ≤ 4 days | 0 | 5 (9.4) | 1 (7.1) | 1 (3.0) |
| Total duration of fever [day] | 11.33±3.29 | 12.13±5.91 | 12.71±5.11 | 14.70±5.81 |
| IVIG resistance | 1 (8.3) | 9 (17.0) | 3 (21.4) | 6 (18.2) |
| Corticosteroid therapy | 1 (8.3) | 4 (7.5) | 1 (7.1) | 4 (12.1) |
| **Laboratory values** | | | | |
| White blood cell count [×10^9^/L, ref.5–12×10^9^/L] | 13.64±3.81 | 14.61±7.03 | 14.67±5.58 | 17.03±8.05 |
| Neutrophilscount [×10^9^/L, ref.1.8–6.3×10^9^/L] | 8.49±5.42 | 8.76±6.02 | 8.91±4.36 | 9.05±7.39 |
| ≥ 80% | 3 (25.0) | 6 (11.3) | 1 (7.1) | 1 (3.0) |
| NLR | 4.02±4.43 | 3.05±3.51 | 3.24±2.74 | 2.23±2.52 |
| Hemoglobin [g/L, ref. 120–160 g/L] | 110.13±8.49 | 106.62±16.48 | 103.03±12.69 | 102.74±15.06 |
| ≤ 110g/L | 8 (66.7) | 30 (56.6) | 8 (57.1) | 22 (66.7) |
| Platelet count [×10^12^/L, ref.125–350×10^12^/L] | 376.30±84.07 | 348.83±211.67 | 376.40±185.80 | 428.82±247.94 |
| PLR | 137.38±114.72 | 116.79±112.17 | 125.71±86.90 | 100.31±71.46 |
| CRP [mg/L, ref. 0–10 mg/L] | 70.46±77.93 | 77.31±63.46 | 79.43±65.29 | 73.49±49.03 |
| ESR [mm/h, ref. 0–20 mm/h] | 56.60±32.66 | 55.28±34.94 | 58.67±30.13 | 64.19±35.88 |
| Sodium [mmol/L, ref. 137–147 mmol/L] | 138.84±2.54 | 135.98±3.05^＊^ | 135.88±2.90 | 137.38±2.79 |
| ≤ 133 mmol/L | 0 | 7 (13.2) | 3 (21.4) | 3 (9.1) |
| ALT [U/L, ref. 7–45U/L] | 23.50 (16.00,92.00) | 25.00 (19.00, 60.50) | 33.00 (20.25, 61.25) | 31.00 (20.00, 72.50) |
| AST [U/L, ref. 13–40U/L] | 44.00 (27.75, 61.25) | 34.00 (26.50, 46.50) | 37.50 (25.75, 59.25) | 29.00 (24.50, 40.00) |
| Total bilirubin [umol/L, ref. 3.4–20.5umol/L] | 6.25 (3.03, 8.50) | 5.50 (3.45, 8.70) | 7.55 (5.83, 13.65) | 4.93 (3.95, 10.15) |
| Albumin [g/L, ref. 40–55 g/L] | 37.83±5.29 | 35.18±5.57 | 32.46±6.80 | 32.41±6.04^＊^ |
| A/G ratio | 1.35±0.41 | 1.48±0.57 | 1.10±0.54 | 1.13±0.59^†^ |
| CRP/albumin ratio | 2.05±2.45 | 2.35±2.03 | 2.82±2.82 | 2.48±1.91 |
| **Maximum Z score** | | | | |
| Baseline | 3.29 (2.78, 4.05) | 3.67 (3.37, 4.62) | 4.47 (3.06, 4.75) | 4.90 (3.54, 6.34)^＊†^ |
| 1 month later | 2.66 (2.57, 3.50) | 3.25 (2.87, 3.77) | 3.72 (3.13, 4.42) | 5.30 (3.53, 6.20)^＊†^ |
| **Outcomes** | | | | |
| Mid- to large-sized CAA | 0 | 10 (18.9) | 3 (21.4) | 19 (57.6)^＊†^ |
| De novo CAA after IVIG treatment | 2 (16.7) | 9 (17.0) | 3 (21.4) | 4 (12.2) |
| Thrombosis | 0 | 1 (1.9) | 0 | 10 (30.3)^†^ |

Data are expressed as means with standard deviations, medians (IQR), or as number (percentage).

*P* value＜0.008 was considered statistically significant after Bonferroni correction for multiple comparion.^＊^statistically significant versus within 2 months group; ^†^statistically significant versus 2–6 months group. CAA, coronary artery aneurysm; BMI, body mass index; KD, Kawasaki disease; IVIG, intravenous immunoglobulin; NLR, neutrophil-to-lymphocyte count ratio; PLR, platelet-to-lymphocyte count ratio; CRP, C-reactive protein; ESR, erythrocyte sedimentation rate; ALT, alanine aminotransferase; AST, aspartate aminotransferase; A/G, albumin-to-globin.

Table 2 Multivariate analysis of candidate risk factors associated with persistence of coronary aneurysms, compared to persistent at > 1 year group

|  | Univariate | | | | | | Multivariate^#^ | | | | | | VIF |
| --- | --- | --- | --- | --- | --- | --- | --- | --- | --- | --- | --- | --- | --- |
|  | within 2 months | | 2-6 months | | 6-12 months | | within 2 months | | 2-6 months | | 6-12 months | |  |
|  | odds ratio (95%CI) | *P* value | odds ratio (95%CI) | *P* value | odds ratio (95%CI) | *P* value | odds ratio (95%CI) | *P* value | odds ratio (95%CI) | *P* value | odds ratio (95%CI) | *P* value |  |
| Sodium | 1.242 (0.951–1.624) | 0.112 | 0.840 (0.713–0.989) | 0.037 | 0.830 (0.665–1.036) | 0.099 | 1.211 (0.829–1.767) | 0.322 | 0.773 (0.624–0.958) | 0.019 | 0.776 (0.591–1.017) | 0.067 | 1.078 |
| Albumin | 1.186 (1.042–1.351) | 0.010 | 1.085 (1.005–1.172) | 0.037 | 1.002 (0.901–1.113) | 0.975 | 1.322 (1.030–1.723) | 0.029 | 1.018 (0.883–1.174) | 0.806 | 1.026 (0.859–1.224) | 0.779 | 2.146 |
| A/G ratio | 2.237 (0.643–7.784) | 0.206 | 3.346 (1.379–8.120) | 0.008 | 0.896 (0.242–3.322) | 0.869 | 0.190 (0.015–2.385) | 0.198 | 3.335 (0.692–16.085) | 0.133 | 0.642 (0.074–5.550) | 0.687 | 2.063 |
| Maximum Z score (baseline) | 0.303 (0.141–0.652) | 0.002 | 0.505 (0.340–0.748) | 0.001 | 0.547 (0.313–0.955) | 0.034 | 0.900 (0.269–3.006) | 0.864 | 1.494 (0.731–3.055) | 0.271 | 1.040 (0.438–2.470) | 0.928 | 2.714 |
| Maximum Z score (1 month later) | 0.122 (0.034–0.435) | 0.001 | 0.357 (0.225–0.556) | ＜0.001 | 0.497 (0.284–0.870) | 0.014 | 0.183 (0.045–0.748) | 0.018 | 0.266 (0.127–0.558) | ＜0.001 | 0.408 (0.175–0.953) | 0.038 | 2.757 |

^#^indicates a significant relationship after correction for age and sex; CI, confidence interval; VIF, variance inflation factors; A/G, albumin-to-globin.
